# Curcumin induces apoptosis in human hepatocellular carcinoma cells by decreasing the expression of STAT3/VEGF/HIF-1α signaling

**DOI:** 10.1515/biol-2022-0618

**Published:** 2023-06-14

**Authors:** Xiaoping Wang, Yu Tian, Huanping Lin, Xiaolan Cao, Zhendong Zhang

**Affiliations:** Key Laboratory of High Altitude Hypoxia Environment and Life Health, School of Medicine, Xizang Minzu University, No. 6 Wenhui East Road, Weicheng District, Xianyang, 712082 Shaanxi, China; Joint Laboratory for Research on Active Components and Pharmacological Mechanism of Tibetan, Materia Medica of Tibetan Medical Research Center of Tibet, School of Medicine, Xizang Minzu University, Xianyang, 712082 Shaanxi, China

**Keywords:** apoptosis, cell cycle, signal transduction, hepatocellular carcinoma

## Abstract

Curcumin is the most abundant derivative of turmeric rhizome. Although studies have proved that curcumin could inhibit the growth of tumors, its specific molecular mechanism has not yet been fully elucidated. This study aims to systematically elaborate the mechanisms of curcumin against hepatocellular carcinoma. The anti-tumor effect of curcumin was determined by the cell viability test. Flow cytometry was applied to examine the cell cycle and the apoptosis of cancer cells, and the cancer cell migration was detected by wound healing experiments. The expressions of signal transducers and activators of transcription 3 (STAT3), vascular endothelial growth factor (VEGF), and hypoxia-inducible factor-1α (HIF-1α) in cancer cells were examined by immunostaining and analyzed by the Image J analysis system. After treatment with curcumin, the apoptosis ratio of HepG2 cells increased significantly (*P* < 0.05). The proliferation of cancer cells was arrested at the S-phase cell cycle, and the migration of cancer cells was inhibited by the increasing concentration of curcumin, together with the decreasing expressions of STAT3, VEGF, and HIF-1α signaling pathways. The results indicate that curcumin could effectively inhibit the growth and migration of hepatocarcinoma cells by inducing cancer cell apoptosis, blocking the cancer cell cycle in the S phase, and reducing the expression of STAT3, VEGF, and HIF-1α signaling pathways.

## 1 Introduction

Hepatocellular carcinoma is the most common malignant gastrointestinal carcinoma around the world [1,2,3]. Although patients with hepatocellular carcinoma can be treated with surgery, radiotherapy, or chemotherapy, the mortality rate is still high [1,2,3]. So it is necessary to seek more effective therapeutic agents against hepatocellular carcinoma. A large amount of food derivatives have exhibited their unique anti-tumor properties, and many studies have confirmed that food derivatives are effective anticancer agents, which has gradually become an important research field in oncology [4,5,6].

Curcumin, a natural polyphenolic compound of *Curcuma longa*, is commonly used as a kind of vegetable, food ingredient, and a traditional herb in Asia, which has proved to possess several biologic activities, such as anti-oxidative, anti-inflammatory, and anti-tumor effects [7,8,9]. Previous studies indicated that curcumin exerts anti-tumor effects on atypical hyperplasia, reversing the metaplasia, decreasing the recurrence of atypical hyperplasia, and improving the life quality of patients [8,9,10]. Compared with chemotherapeutic agents, curcumin is less toxic and more compatible with biological tissues; therefore, it deserves further study on its effects against hepatocellular carcinoma [7,8,9,10].

Apoptosis plays a crucial role in the proliferation and turnover of cells in various tumors [11,12,13]. Research indicated that curcumin may elicit apoptosis of cancer cells [14,15,16]. Curcumin is likely to inhibit hepatocellular carcinoma cell proliferation and cause tumor cell death. Moreover, curcumin demonstrates its anti-tumor effects by disturbing several signaling pathways, such as ras, AKT, monoamine oxidase A/mammalian target of rapamycin (mTOR)/hypoxia-inducible factor-1α (HIF-1α), signal transducers and activators of transcription 3 (STAT3), and vascular endothelial growth factor (VEGF) pathways [17,18,19,20]. These results suggested that curcumin could regulate numerous signaling molecules and exert anti-tumor effects. Therefore, curcumin may inhibit the growth of tumors through numerous signaling pathways.

HIF-1α has been proved to participate in the transcription of downstream target genes, such as VEGF, erythropoietin, and glucose transporter 1 gene, and activate multiple signaling pathways that lead to tumor cell proliferation, angiogenesis, invasion, and metastasis [21,22]. The research found that colon cancer, breast cancer, gastric cancer, and renal cell carcinoma overexpressed HIF-1α [23,24,25]. Studies also showed that the expression of HIF-1α was significantly correlated with the progression of hepatocellular carcinomas [26,27]. JAK/STATs signaling pathway is closely related to cell proliferation, differentiation, and apoptosis, which can lead to abnormal proliferation and malignant transformation [28,29]. Research confirmed that there was a high expression of STAT3 in hepatocellular carcinoma, which was closely related to metastasis and tumor grades [30]. Tumor angiogenesis is the basis of tumor growth and metastasis. VEGF is known as the most important and specific angiogenic factor, which promotes angiogenesis and tumor cell proliferation by binding vascular and lymphatic endothelial cell surface receptors and tumor cell surface receptors through the autocrine pathway [31,32,33]. Studies showed that the overexpression of STAT3 and VEGF in tumor cells can increase the microvessel density and promote the progression of hepatocellular carcinoma [34,35,36]. The high expression of VEGF in tumor tissue in patients with hepatocellular carcinoma is closely related to a poor prognosis [35,36,37,38]. Based on the previous findings, it is presumed that the high expression of HIF-1α in hepatocellular carcinoma tissues is positively correlated with STAT3 and VEGF. The three signaling molecules formed the HIF-1α/STAT3/VEGF signal transduction pathways, which played an important role in the occurrence and development of hepatocellular carcinoma.

In the present study, we investigated the mechanisms of curcumin against hepatocellular carcinoma and examined whether curcumin could interfere with the HIF-1α/STAT3/VEGF signal transduction pathways, induce apoptosis, and arrest the cell cycle against the proliferation of cancer cells, further confirming the multiple anti-tumor bioactivities of curcumin.

## 2 Materials and methods

### 2.1 Antibody reagents and cell line

HepG2 human hepatocellular carcinoma cells were kindly provided by School of Medicine, Xi’an Jiaotong University, and maintained in RPMI 1640 (Gibco-BRL, USA) with 10% fetal bovine serum, 100 U/ml penicillin, and 100 μg/ml streptomycin (Invitrogen Corp., CA, USA) at 37°C under a humidified atmosphere of 95% air and 5% CO_2_.

Rabbit anti-human cyclin-A1, cyclin-B1, Bcl-2, Bax, Caspase-3, HIF-1α, STAT3, and VEGF polyclonal antibodies were purchased from Santa Cruz Biotechnology, Inc. (Santa Cruz, CA, USA). EnVisionTM kits were purchased from Dako Corp. (Carpinteria, CA, USA).

### 2.2 Preparation of curcumin solution

Curcumin was purchased from Sigma (MO, USA). Curcumin was dissolved in dimethyl sulfoxìde (DMSO) at the stock concentration of 10 mmol/l, then stored at −20℃. This is the basic solution, which can be diluted in phosphate buffered saline (PBS) as needed, and the percentage of DMSO in solutions injected into mice was 0.1%.

### 2.3 Cell viability assay

HepG2 cells (4 × 10^3^) were seeded in a 96-well plate. After an overnight cultivation, cells were treated with different concentrations of curcumin for 6, 12, 24, 48, and 72 h. The non-radioactive cytotoxicity lactate dehydrogenase release assay kit (Promega, USA) was used to measure the cytotoxicity of curcumin against cancer cells in concentrations of 10, 20, 30, 40, 50, 60, and 80 μmol, according to the manufacturer’s protocol. Specific lysis was calculated according to the following formula: percent specific lysis = [(experimental release value − effector spontaneous release value − target spontaneous release value)/(target maximum release value − target spontaneous release value)] × 100%. The results shown are representative of experiments repeated three times.

### 2.4 Detection of apoptosis

HepG2 cells were detached with 0.25% trypsin, and the cell density was adjusted to 1 × 10^6^/ml, then cultivated with curcumin in concentrations of 20, 40, and 60 μmol for 48 h. The next day, cancer cells were collected to detect apoptosis.

For the detection of apoptotic cells, apoptotic rates were examined by flow cytometry analysis. Annexin V-fluoresceine isothiocyanate (FITC) and propidium iodide (PI) staining were used for flow cytometry detection of apoptosis. About 1 × 10^6^ cells from each sample were treated with RNase and stained with Annexin V-FITC and PI. The apoptotic cells with DNA strand breaks that had been labeled were measured on a flow cytometer (FACSCalibur, Becton Dickinson, USA). The data from 10^6^ cells/sample were collected, stored, and analyzed using CELLQUEST (Becton Dickinson, USA) and ModFIT LT for mac V1.01 software (Becton Dickinson).

### 2.5 Cell cycle analysis

To determine the effect of curcumin on the cell cycle, HepG2 cells were seeded at a density of 3 × 10^5^ cells/well in six-well plates and incubated at 37℃ overnight. Then, the cells were exposed to 20, 40, and 60 μmol curcumin for 48 h. Cells were collected, fixed with ice-cold 70% (v/v) ethanol, and kept at 4℃ overnight. Thereafter, cells were collected and washed with PBS. The cell pellets were re-suspended and stained in PBS containing 0.1 mg/ml RNase I and 50 mg/ml PI for 30 min at room temperature. Cell distribution across the cell cycle was determined with a FACScalibur flow cytometer (BD, USA).

### 2.6 Wound healing assay

HepG2 cells were seeded in six-well plates at a concentration of 2 × 10^6^ cells per well and incubated at 37℃ overnight. Cell monolayers that converged almost 100% were wounded with a sterile 20 μl pipette tip. Remove detached cells from the plates carefully with PBS and add RPMI 1640. Cancer cells were stimulated with the different doses of curcumin. After the incubation for 48 h, medium was replaced with PBS, and the scratched areas were photographed using an Olympus microscope. The migration rate was calculated as follows: percent migration rate = (initial area − residual area/initial area) × 100%. Independent experiments were repeated in triplicate.

### 2.7 Immunostaining methods

The slides of cells were fixed with ice-cold 70% (v/v) ethanol for 30 min, then rinsed with PBS. Endogenous peroxidase was then blocked with 3 ml/l H_2_O_2_ diluted in methanol for 30 min at room temperature. Antigen retrieval was performed by treating the slides in citrate buffer in a microwave for 10 min. The slides were incubated in a moist chamber with cyclin-A, cyclin-B1, Bcl-2, Bax, Caspase-3, HIF-1α, STAT3, or VEGF rabbit polyclonal antibody (1:100), respectively, at 4℃ overnight. After a complete wash in PBS, the slides were incubated with horseradish peroxidase-labeled goat anti-mouse antibody (1:100) for 45 min at 37℃. After a complete wash in PBS, the slides were developed in 0.5 g/L freshly prepared 3,3′-diaminobenzedine solution (Sigma Co., St Louis, MO, USA) for 8 min, then counterstained with hematoxylin, dehydrated, air dried, and mounted. Normal human albumin was used to substitute for the primary antibody as a negative control. Only distinctive intranuclear or intra-cytoplasm immunoreactivity was considered positive. In each case, more than 1,000 cells were counted, and the percentage of immunoreactivity was independently determined. Image J analysis software was used to acquire the optical density (OD) data from the stained sections.

### 2.8 Statistical analysis

All data are presented as mean value ± SEM. Statistical analysis and graphical representation of the data were evaluated using GraphPad Prism 5.0 (GraphPad Software, San Diego, CA). Statistical significance was evaluated using Student’s *t*-test, analysis of variance with the least significant difference post hoc test, or *χ* ^2^ test, as appropriate. *P* < 0.05 was considered statistically significant.

## 3 Results

### 3.1 Curcumin suppressed the growth of hepatocarcinoma cells

**Figure 1:**
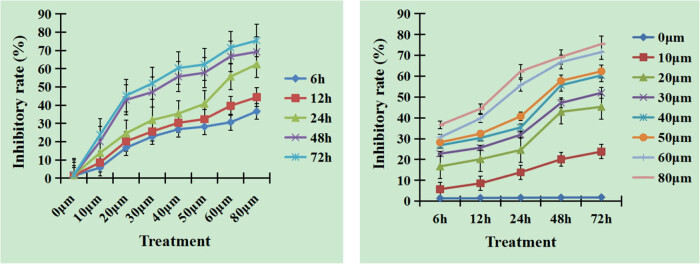
Inhibition effect after different doses of curcumin treatment. Curcumin suppressed tumor cell growth with the change of times and concentrations. The inhibitory rates increased with the time extension and the higher concentrations of curcumin. The differences between curcumin treatment groups were significant, *n* = 8, *P* < 0.05.

To verify the proliferation inhibition of curcumin against hepatocellular carcinoma cells, we detected cell viability. Compared with the cell culture medium control group, tumor cell proliferation was significantly inhibited in the different concentrations of curcumin. The inhibitory rates of HepG2 cells were increased with the time extension and the higher concentrations of curcumin, Figure 1. The results indicate that the proliferation inhibitory effect of curcumin on HepG2 cells is time- and dose-dependent, and the differences between curcumin treatment groups are significant.

### 3.2 Curcumin induced the apoptosis of hepatocarcinoma cells

**Figure 2:**
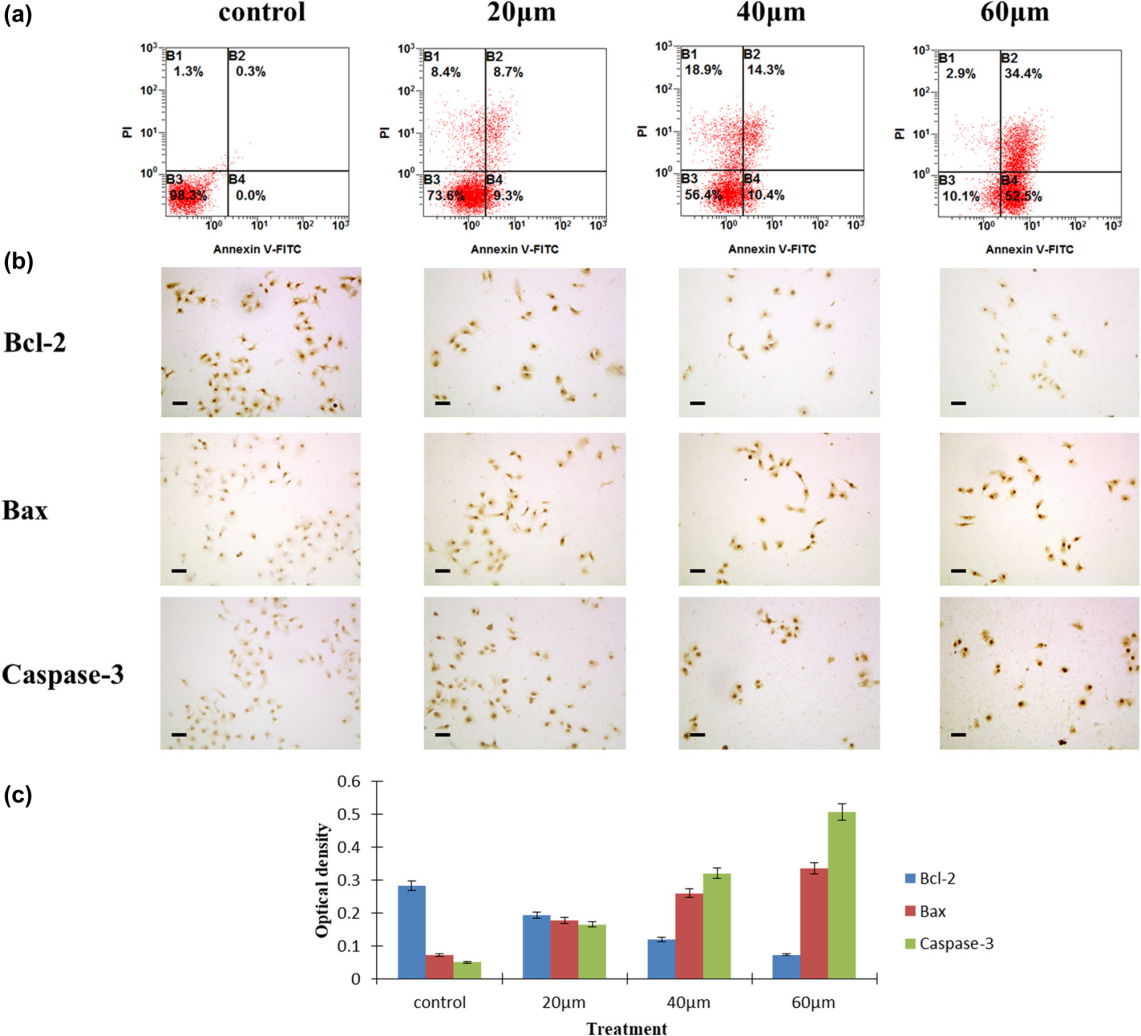
Curcumin elicited the apoptosis in cancer cells. (a) Apoptosis rate in HepG2 cells examined by flow cytometry. (b) Expressions of Bcl-2, caspase-3, and Bax in cancer cells by immunostaining analysis. The OD value (c) of the expressions of Bcl-2, caspase-3, and Bax in the experimental groups and control group of HepG2 cells after curcumin treatment, *n* = 4.

After being cultured with different concentrations of curcumin, hepatocellular carcinoma cells showed significantly elevated apoptotic percentage in concentrations of 20, 40, and 60 μmol curcumin treatment groups, compared with the control group from 18.0%, 24.7%, 86.9% vs 0.3% shown in FACScan assay, *P* < 0.05, Figure 2a. In order to elucidate the mechanisms of curcumin eliciting the apoptosis of HepG2 cells, we detected the expressions of the apoptotic proteins Bcl-2, Bax, and caspase-3 by immunoreactivity and Image J software analysis. Bcl-2, Bax, and caspase-3 were mainly stained in the cell cytoplasm. With the increasing curcumin concentrations, the expression of the anti-apoptotic protein Bcl-2 was downregulated, while the expressions of the apoptotic proteins Bax and caspase-3 were upregulated, Figure 2b. The OD values of Bcl-2 in curcumin treatment groups were significantly lower than those in the control group, *P* < 0.05, Figure 2c, while the OD values of Bax and caspase-3 in curcumin treatment groups were higher than those in the control group, *P* < 0.05, Figure 2.

### 3.3 Curcumin arrested the cell cycle of hepatocarcinoma cells

**Figure 3:**
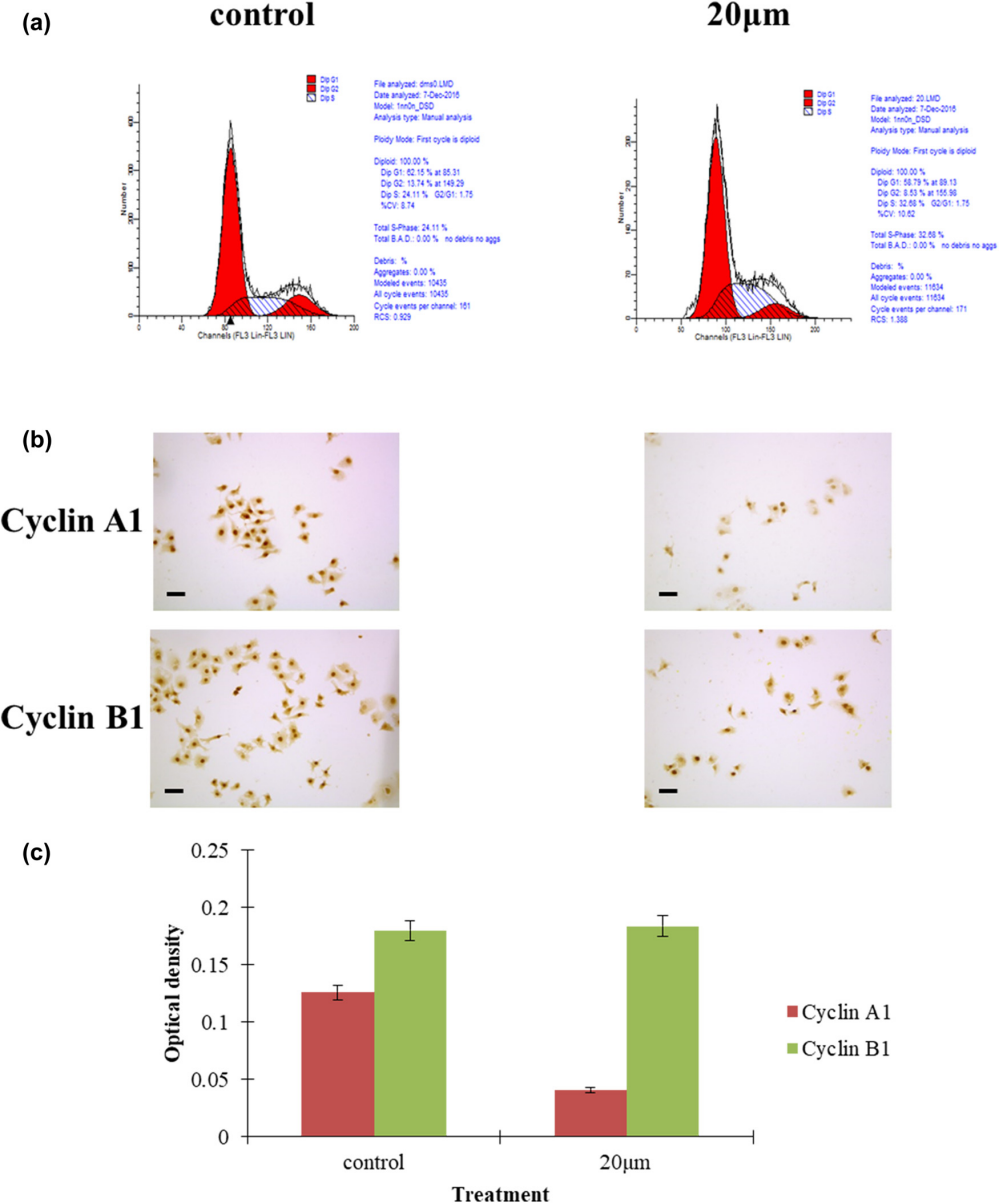
Curcumin elicited hepatocellular carcinoma cell cycle arrest. (a) The cell cycle S-phase was arrested in cancer cells with 20 μmol curcumin treatment for 48 h. (b) Immunostaining of cyclin B1 and cyclin A1 in cancer cells treated with curcumin, bars 20 μm. (c) The OD value of cyclinA1 measured in the experimental group after 20 μmoL curcumin treatment was lower than that in the control group.

In order to confirm the cell cycle arrest of curcumin on hepatocellular carcinoma cells, the cancer cells treated with curcumin were analyzed by flow cytometry and immunostaining of cyclin A1 and cyclin B1. After being cultured with low-dose curcumin, hepatocellular carcinoma cells were significantly arrested at the S-phase cell cycle, as shown in the FACScan assay, Figure 3a. The low-dose curcumin treatment group displayed S-phase cell cycle arrest, which was further verified by the low expression of cyclin A1, Figure 3b. The OD value of cyclin A1 in curcumin treatment groups was lower than that in the control group, *P* < 0.05, while the OD value of cyclin B1 in the 20 μmol curcumin treatment group was similar to that in the control group, *P* > 0.05, Figure 3c. It seemed that curcumin could inhibit hepatocellular carcinoma cell proliferation by downregulating the expression of cyclin A1. However, the expression levels of cyclin B1 remained unchanged, Figure 3c.

### 3.4 Curcumin suppressed the migration of hepatocarcinoma cells

**Figure 4:**
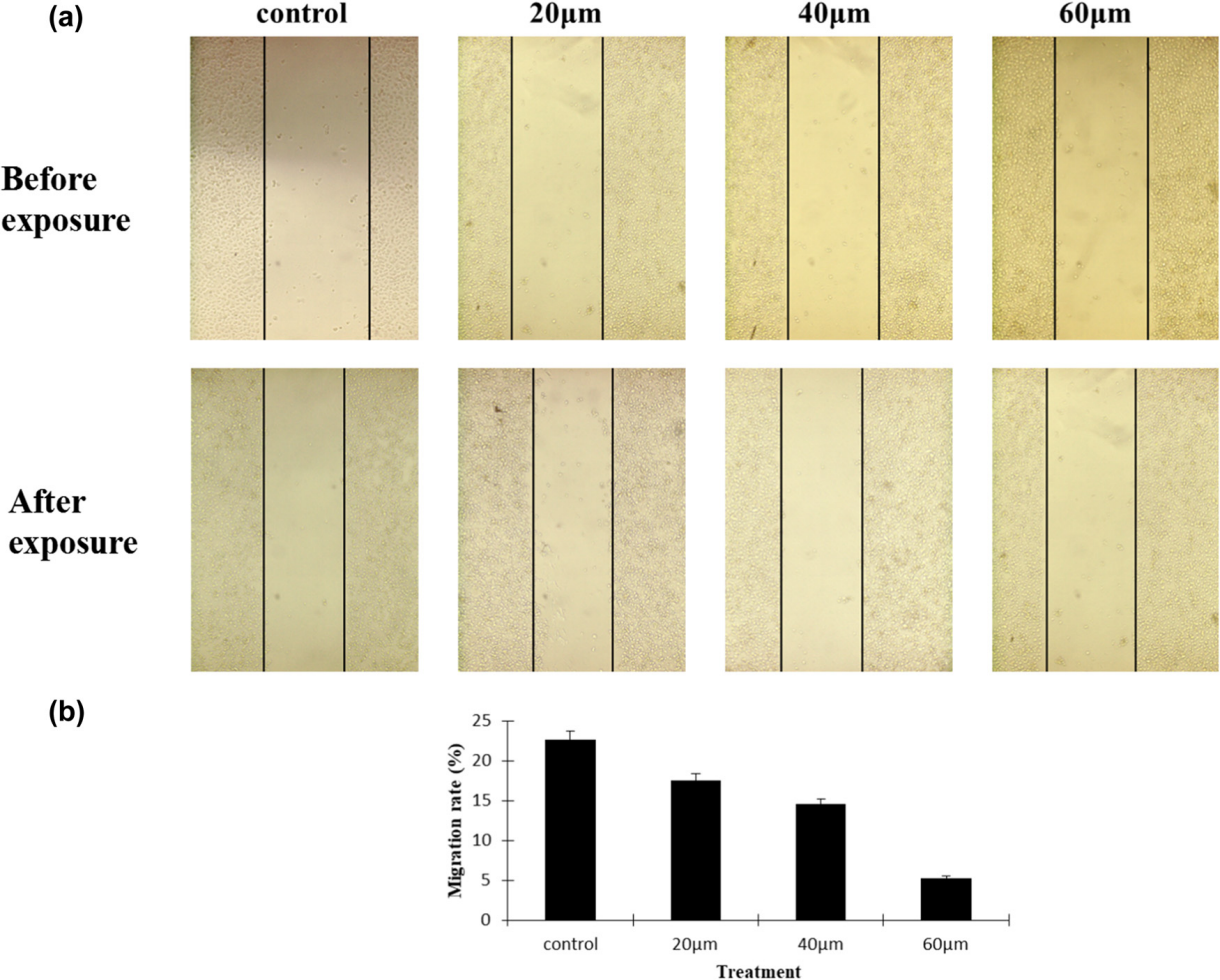
Curcumin suppressed cancer cell migration. (a) The HepG2 cancer cell migration inhibition effect was determined by a wound healing assay. The concentration of curcumin is proportional to the cancer cell migration inhibition; as the concentration increased, the level of cancer cell migration decreased. (b) Curcumin treatment groups vs the control, *n* = 4, *P* < 0.05.

As the ability of cancer cells to migrate is regarded as one of the pivotal processes in the development of tumor invasion, a wound healing assay was performed to verify the contribution of curcumin to the migration potential of cancer cells. The results demonstrated that curcumin treatment significantly led to reduced wound closure in HepG2 cells. Moreover, curcumin inhibited HepG2 cells motility in a dose-dependent manner, Figure 4a. In accordance with the wound closure, curcumin treatment resulted in decreased migration rates of HepG2 cells, *P* < 0.05, Figure 4b. Taken together, curcumin indeed exerts its inhibitory effect on HepG2 cell motility.

### 3.5 Curcumin suppressed the expression of STAT3, VEGF, and HIF-1α in hepatocarcinoma cells

**Figure 5:**
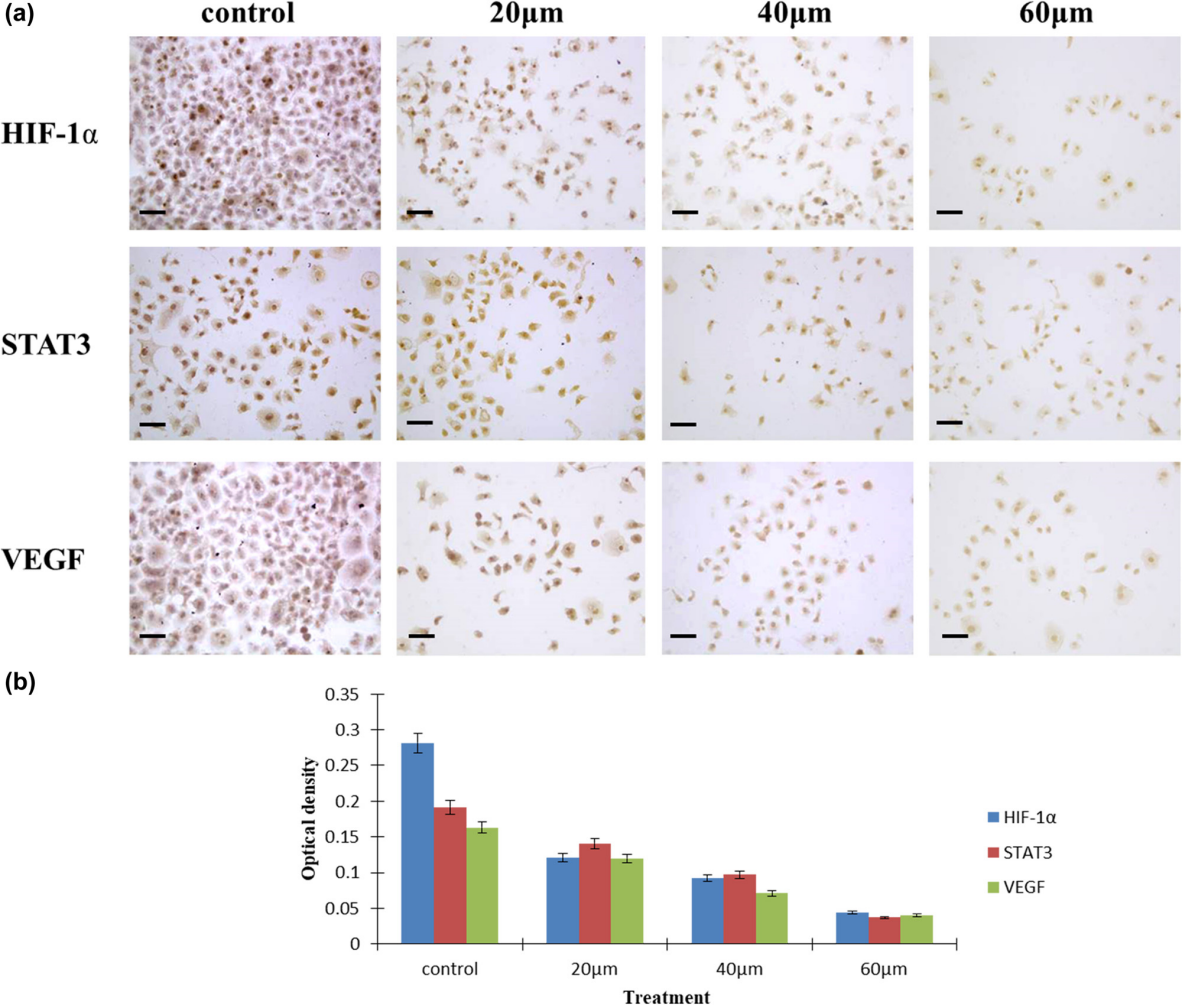
Expression of STAT3, VEGF, and HIF-1α in HepG2 cells. (a) The locations of STAT3, VEGF, and HIF-1α in hepatocarcinoma cells. (b) The OD values of STAT3, VEGF, and HIF-1α signaling pathways measured after curcumin treatment. In the experimental groups, the OD values of STAT3, VEGF, and HIF-1α were lower compared with the control, *n* = 4, *P* < 0.05.

To determine whether curcumin affects the expressions of HIF-1α, STAT3, and VEGF signaling pathways in hepatocellular carcinoma cells, we examined the expressions of the above signal molecules by immunoreactivity and Image J software analysis. HIF-1α and STAT3 proteins were mainly stained in the cell nucleus, while VEGF was mainly localized in the cell cytoplasm, as shown in Figure 5a. The OD values of HIF-1α, STAT3, and VEGF in curcumin treatment groups were significantly lower than those in the control group, *P* < 0.05, Figure 5b.

## 4 Discussion

It was found that curcumin, the main component of *Curcuma longa*, could suppress the proliferation of various cancers by inducing apoptosis, arresting the cell cycle, and altering several important signal transduction pathways [12,13,14,15,16]. Since it possesses multiple functions and lower toxic side effects, curcumin has been under investigation for clinical trials [39,40]. Therefore, we presumed that curcumin should have multiple effects against hepatocellular carcinomas, and it is necessary to conduct a profound research to elucidate the anti-tumor mechanisms of curcumin. In the present study, we showed that curcumin inhibited the proliferation of HepG2 cells. We found that the time- and dose-dependent anti-tumor effects of curcumin were caused by inducing apoptosis of tumor cells, arresting the cell cycle, and inhibiting the proliferation of tumor cells. We further confirmed that curcumin could downregulate the expression of the signal transduction pathways HIF-1α/STAT3/VEGF.

Compared with the cell culture medium control group, tumor cell proliferation was significantly inhibited in the different concentrations of curcumin. The inhibitory rates of HepG2 cells increased with the time extension and the higher concentrations of curcumin. The results indicated that the proliferation inhibitory effect of curcumin on HepG2 cells was time- and dose-dependent, and the differences between curcumin treatment groups were significant.

After being cultured with different concentrations of curcumin, hepatocellular carcinoma cells showed a significantly elevated apoptotic percentage with the increasing concentrations of curcumin in the treatment groups. In order to elucidate the mechanisms of curcumin eliciting the apoptosis of HepG2 cells, we detected the expression of the apoptotic proteins Bcl-2, Bax, and caspase-3. With the increasing curcumin concentrations, the expression of the anti-apoptotic protein Bcl-2 was downregulated, while the expressions of the apoptotic proteins Bax and caspase-3 were upregulated.

Similar to other malignant tumors, hepatocellular carcinoma is always with heterogeneous cell proliferation and differentiation, as well as abnormal apoptosis [41,42]. The increased induction of apoptosis in hepatocellular carcinoma cells can be detected after treatment with chemotherapeutic agents and some herbs [42,43,44]. These data suggest that inducing the apoptosis of tumor cells should be a therapeutic mechanism for hepatocellular carcinoma. The present study indicated that tumor growth was significantly suppressed by treatment with curcumin. Flow cytometry analysis showed that curcumin could enhance the apoptosis of hepatocellular carcinoma cells. The results are consistent with the findings of curcumin recently reported in other cancer research [45,46]. Our results suggest that an important mechanism of curcumin inhibiting proliferation of hepatocellular carcinoma cells is to elicit apoptosis, which is in agreement with the downregulation of the anti-apoptotic protein Bcl-2 and the upregulation of the apoptotic proteins Bax and caspase-3.

The low-dose curcumin treatment group displayed S-phase cell cycle arrest, which was further verified by the low expression of cyclin A1. It seemed that curcumin could suppress hepatocellular carcinoma cell proliferation via downregulating the expression of cyclin A1. However, the expression levels of cyclin B1 remained unchanged. Our results are different in some way from most of the studies in which curcumin is a potent agent to arrest cell cycle at G2/M phase [47,48,49,50,51]. Interestingly, several research indicated that curcumin inhibited the cell cycle according to the various tumor histological types [47,48,49,50]. Some research revealed that curcumin induces G2/M arrest in oral carcinoma, non-small cell lung cancer, breast cancer, pancreatic cancer, and renal carcinoma [48,49,50,51]. Few studies reported that cell cycle arrest in colon cancer cells occurred at the S phase [52,53]. Our results suggested that curcumin led to S-phase arrest in HepG2 cells. The treatment of hepatocellular carcinoma cells with curcumin resulted in an increase in the relative proportion of cells in the S phase. We further explored the underlying molecular mechanism of curcumin-induced S-phase arrest. To this end, we analyzed the expressions of cyclin A1 and B1, the pivotal regulatory proteins of the S and G2/M transitions. Curcumin-treated HepG2 cells exhibited diminished expression of cyclin A1, while the expression of cyclin B1 remained relatively constant in all tested conditions. We thus presumed that curcumin instigates cell cycle arrest by downregulating the expression of cyclin A1. Although low-dose curcumin could not induce the higher apoptosis of cancer cells, it was able to arrest the tumor cell cycle in the S-phase, which may account for the proliferation suppression and late apoptosis of cancer cells.

Several research showed that curcumin could inhibit hepatocarcinoma cell migration and invasion by inhibiting transforming growth factor-β1-induced epithelial-mesenchymal transitions, the phosphorylation of Smad4, Src, or p38, and decreasing the expression and activity of matrix metalloproteinases (MMP)-2 and MMP-9 [54,55,56]. Recent reports indicated that curcumin suppressed the migration of lung cancer cells by disturbing the signal transduction pathways PI3K/AKT/mTOR [57]. Our results indicate that curcumin suppressed hepatocellular carcinoma cell migration by disturbing the cell cycle and transduction signal pathways HIF-1α/STAT3/VEGF.

To determine whether curcumin affects the expressions of HIF-1α, STAT3, and VEGF signaling pathways in hepatocellular carcinoma cells, we examined the expressions of the above signaling pathways. Signal transduction pathways play important roles in the proliferation and development of cancer cells [18,19,20,21,22]. HIF-1α mediates hypoxic and non-hypoxic signaling pathways and plays a crucial role in cancer progression [21,22,23,24,25]. Overexpression of HIF-1α has been found in many gastroenterological cancers, which is implicated in drug resistance and poor prognosis of cancers [21,22,23,24,25]. The JAK/STAT3 signal cascade exhibits essential roles in promoting cancer cell survival, proliferation, angiogenesis, and tumor metastasis [28,29,30]. STAT3 signaling is activated in a variety of tumors to inhibit apoptosis, increase proliferation, and thus promote the initiation and development of tumors [31,32,33,34,35]. Studies showed that HIF-1α could also combine with STAT3, working together on the downstream genes when they were induced by cytokines [58,59]. The inappropriate activation of the two transcription factors may lead to cell transformation, promoting the development of hepatocellular carcinomas [58,59]. Tumor angiogenesis is a multi-step vascular remodeling of the extracellular matrix, basement membrane degradation, endothelial cell migration, proliferation, and neovascularization. VEGF has been proved to be a major inducer of angiogenesis, lymphangiogenesis, and vasculogenesis in cancers [33,34,35]. The high expression of VEGF in serum and tumor tissues in patients with hepatocellular carcinoma is closely related to poor prognosis [33,34,35,60]. It was verified that the upregulation of HIF-1α, STAT3, and VEGF in tumor cells was likely to enhance the microvessel density and promote the progression of some cancers [36,60,61]. On the basis of the previous findings, we deduced that the high expression of HIF-1α in hepatocellular carcinoma cells is positively correlated with STAT3 and VEGF. HIF-1α, STAT3, and VEGF signaling molecules interact and form the HIF-1α/STAT3/VEGF signal transduction pathway, which probably plays an important role in the occurrence and development of hepatocellular carcinoma. Some studies showed that curcumin could inhibit the proliferation and progression of several cancers by decreasing the expression of HIF-1α, STAT3, or VEGF signaling molecules, as well as inducing cell apoptosis [39,40,62]. At present, few studies confirmed the effect of curcumin on the HIF-1α/STAT3/VEGF signal transduction pathway against hepatocellular carcinoma. Our study clarified that curcumin could downregulate the expressions of HIF-1α/STAT3/VEGF signal transduction pathway in hepatocellular carcinoma, thus suppressing the proliferation and growth of the cancer cells.

The present study verified that curcumin could inhibit the growth of hepatocellular carcinoma cells, and the main mechanisms are apoptosis induction, cell cycle arrest, and downregulation of the expression of cell signal transduction pathways HIF-1α/STAT3/VEGF. The findings indicate that curcumin exerts multiple anti-tumor effects for inhibiting the tumor growth, which laid foundation for future clinical trials. However, the underlying molecular mechanisms through which curcumin exerts its anti-tumor activities still need further investigation.
